# A Closer Look at Histamine in *Drosophila*

**DOI:** 10.3390/ijms25084449

**Published:** 2024-04-18

**Authors:** Cinzia Volonté, Francesco Liguori, Susanna Amadio

**Affiliations:** 1 National Research Council, Institute for Systems Analysis and Computer Science “A. Ruberti”, Via Dei Taurini 19, 00185 Rome, Italy; f.liguori@hsantalucia.it; 2 Experimental Neuroscience and Neurological Disease Models, Santa Lucia Foundation IRCCS, Via Del Fosso di Fiorano 65, 00143 Rome, Italy; s.amadio@hsantalucia.it

**Keywords:** carcinine, circadian rhythm, courtship behavior, histidine decarboxylase, histamine receptor, histamine transporter, mechanosensory transmission, photoreceptor, temperature sensing, visual transmission

## Abstract

The present work intends to provide a closer look at histamine in *Drosophila*. This choice is motivated firstly because *Drosophila* has proven over the years to be a very simple, but powerful, model organism abundantly assisting scientists in explaining not only normal functions, but also derangements that occur in higher organisms, not excluding humans. Secondly, because histamine has been demonstrated to be a pleiotropic master molecule in pharmacology and immunology, with increasingly recognized roles also in the nervous system. Indeed, it interacts with various neurotransmitters and controls functions such as learning, memory, circadian rhythm, satiety, energy balance, nociception, and motor circuits, not excluding several pathological conditions. In view of this, our review is focused on the knowledge that the use of *Drosophila* has added to the already vast histaminergic field. In particular, we have described histamine’s actions on photoreceptors sustaining the visual system and synchronizing circadian rhythms, but also on temperature preference, courtship behavior, and mechanosensory transmission. In addition, we have highlighted the pathophysiological consequences of mutations on genes involved in histamine metabolism and signaling. By promoting critical discussion and further research, our aim is to emphasize and renew the importance of histaminergic research in biomedicine through the exploitation of *Drosophila*, hopefully extending the scientific debate to the academic, industry, and general public audiences.

## 1. Histamine in *Drosophila*

**Figure 1:**
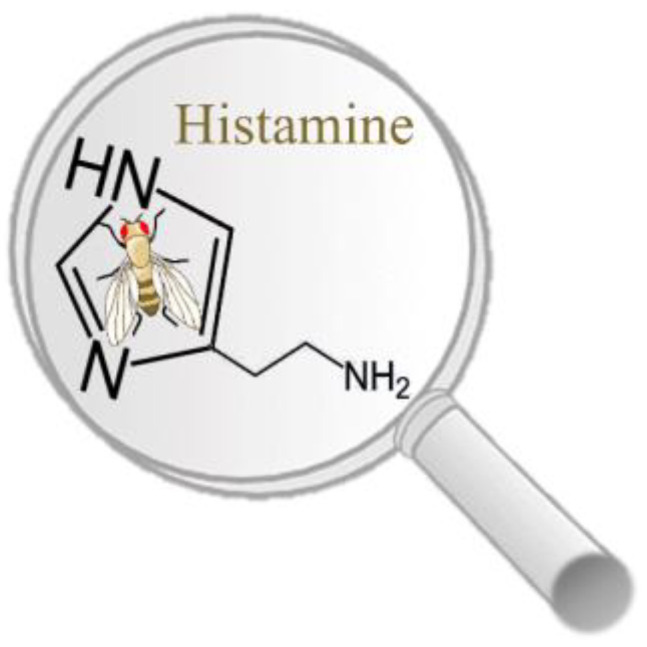
A closer look at histamine in *Drosophila*.

*Drosophila melanogaster*, or the fruit fly, has been widely used for more than a century in biological research, and its use in genetics, physiology, pathology, and evolution goes back to C.W. Woodworth, the very first to breed the fruit fly in captivity and to propose *Drosophila* as a model organism for scientific exploitation. Seminal discoveries were made with the aid of this invertebrate, such as the chromosomal theory of inheritance [1] that, back in 1933, gained Nobel prize recognition for T.H. Morgan. Five additional Nobel prizes have been awarded since then for groundbreaking discoveries made using fruit flies, such as the demonstration that X-ray irradiation increases mutation rates, the genetic control of embryonal development, the odor receptors and organization of the olfactory system, the activation of innate immunity, and the molecular mechanisms of circadian rhythms. Today, more than 120,000 or 30,000 publications are listed in PubMed searches with the basic queries “*Drosophila*” or “*Drosophila* model”, respectively. This emphasizes the high impact of this organism, not just as a genetic box to disentangle basic mechanisms and pathways, but also, above all, as a versatile experimental organism to model normal and/or pathological conditions. The limitations and uncertainties are few indeed, but there are several advantages of exploiting the fruit fly in biomedical research. This is the main reason why *Drosophila* has attracted massive scientific interest and poses several challenges in the histaminergic field (Figure 1).

Histamine is a nitrogenous hydrophilic compound identified in nature, but also chemically synthesized, whose physiological functions in immune responses and neurotransmission were first described at the beginning of the previous century [1]. Since then, we have progressively gained clear evidence on the type of impact that histaminergic research has demonstrated for human health over the years, for instance, by considering that the identification of antagonists for histamine receptors has gained the recognition of two Nobel prizes in 1957 to D. Bovet (for the first synthesis of antihistamines) and in 1988 to J. Black (for the development of the H2R antagonist cimetidine).

Unlike the adult vertebrate brain, where histaminergic neurons are confined to the posterior hypothalamic area in the tuberomammillary nucleus [2,3], the pattern of histamine expression in the adult *Drosophila* is more complex. It comprises retinal photoreceptors in the central nervous system (CNS), in addition to mechanosensory neurons present in bristles and pegs from hair sensilla on the entire body’s surface [4,5] in the peripheral nervous system (PNS). Additionally, histamine immunoreactivity is found in the extraretinal eyelet photoreceptor axons [6], in ocelli, as well as in 18 neurons in the protocerebrum and 2 maxillary–labial neurons with terminations in the subesophageal ganglion, which arborize extensively in non-glomerular neuropil [7,8], and finally in the olfactory system [9], and in putative neurosecretory cells and interneurons innervating the body ganglia [10]. Histamine immunoreactivity is instead apparently absent from the clock neurons and from the chemosensory neurons of adult flies [4,5]. In the larva, histamine-positive labeling is detected in three clustered non-photoreceptor neurons and two additional neurons that establish arborizations with the subesophageal region in each brain hemisphere, but without overlapping with putative afferent gustatory terminals in the larval antennal lobe, and in the tritocerebtrum–subesophageal ganglion region (devoted to decoding mechanosensory and gustatory sensory signals from the cuticular hair sensilla of the head, mouth, and trunk) [9]. Chemosensory processing is histamine-independent, not only in adults, but also in larvae, and specific histamine staining is therefore absent from the chemosensory neurons of the dorsal organ (the larval antenna) and terminal organ, or from the larval antennal lobe and chemosensory local interneuron and projecting afferent neurons. Histamine-positive signals are instead found in some ventral nerve cord neurons identified as putative precursors of histamine–immunopositive cells of the adult thoracic CNS [5,11].

## 2. Histamine Receptors in *Drosophila*

In *Drosophila* and in insects in general, histamine acts as a neurotransmitter mainly for photoreception and mechanosensitivity, but also affects functions such as the circadian rhythm, temperature sensing, and courtship behavior. In the visual system of fruit flies, histamine is synthesized and stored in retinal photoreceptor neurons, undergoing Ca^2+^-dependent release, for instance, after light stimuli. Once released, histamine receptor occupancy on the postsynaptic membrane directly opens histamine-gated chloride channels, causing an inward chloride flux that induces hyperpolarization, thus increasing the threshold for action potentials and membrane currents in postsynaptic interneurons [12,13,14,15,16,17]. The opening of the channel mediates fast synaptic transmission between presynaptic photoreceptor neurons in the retina and postsynaptic first-order interneurons (large monopolar cells) in the lamina (a special interface structure for visual efficiency) within the optic lobe. Details about the structural organization of the visual system are provided below (Section 4.1).

As established by the *Drosophila* genome project [18], two distinct histamine-gated channel genes were predicted to contribute to this specific ionotropic chloride channel function. They are *hclA* (also known as *ort*—*ora transientless*, *hisCl-α1*, *hisCl2*, or *HA-Cl I* by different research groups) and *hclB* (also known as *hisCl1*, *hisCl-α2*, or *HA-Cl II*) [19]. The two channels possess 51% identical amino acids and are related to the *Drosophila* glutamate- and γ-aminobutyric acid (GABA)-gated chloride channels. They both belong to the Cys-loop receptor superfamily characterized by four membrane-spanning domains, sharing about 40% identity with mammalian glycine receptor subunits. Both Ort and HisCl1 (as named from now on) are, however, very different from the receptors identified in vertebrates, which are classified as H1R, H2R, H3R, and H4R (respectively encoded by the *HRH1*, *HRH2*, *HRH3*, and *HRH4* genes in humans), and they are 7-transmembrane rhodopsin-like G protein-coupled metabotropic receptors. Expression profiling confirms that *ort* is expressed in large monopolar interneurons of the lamina, while *hisCl1* expression in the lamina is restricted exclusively to glial cells [15,19]. In particular, the *ort* receptor mRNA is found to be natively expressed in the lamina of the *Drosophila* eye, thus reinforcing its involvement in the visual system. Moreover, RT-PCR analysis has demonstrated the presence of both *ort* and *hisCl1* in the late pupal stage and in the adult head and body of *Drosophila*, but the receptors seem to be absent from the eggs and larvae. In situ hybridization has furthermore confirmed the localization of *ort* (but not of *hisCl1*) throughout the entire lamina neuropil [15].

When expressed in oocytes, Ort forms functional, homomeric receptors with EC50 for histamine = 166 ± 12 μM and Hill coefficient = 1.9 ± 0.3. The prolonged application of histamine causes weak desensitization. The receptor does not respond to the specific vertebrate metabotropic H1R and H3R agonists dimaprit and R-α-methylhistamine. In the presence of 150 μM histamine, the competitive antagonists d-tubocurarine (acting at nicotinic acetylcholine receptors), cimetidine (acting at vertebrate H2R), and pyrilamine (inverse agonist at vertebrate H1R) reversibly block the current induced by Ort with a median IC50 = 3.5, 117, or 165 μM, respectively [15]. Moreover, cimetidine and hydroxyzine (vertebrate H1R antagonist) can effectively inhibit the receptor in vivo and ex vivo, and modulate vigilance states by increasing rest time and reducing sleep latency in the fruit fly [20].

When expressed in oocytes, functional homomeric HisCl1 becomes more sensitive to histamine than Ort, with EC50 = 10.8 ± 0.46 μM and Hill coefficient = 1.7 ± 0.2. Moreover, in the presence of 10 μM histamine, the antagonists d-tubocurarine, cimetidine, and pyrilamine reversibly block the receptor, with IC50 = 5.1, 21, and 442 μM, respectively. Dimaprit (1 mM) does not activate, but acts as an antagonist at the HisCl1 receptor (IC50 = 56 ± 9.6 μM). R-α-methylhistamine (a potent H3R agonist) serves as a partial agonist (EC50 = 202 ± 21 μM) [15].

Taking advantage of one of the most commonly used macrophage-like fly cell lines, *Drosophila* Schneider S2 cells transfected with histamine chloride channels, it was further reported that both the homomeric and heteromeric forms of the receptors are expressed. Ort homomers have almost identical histamine sensitivity (EC50 = 25 μm) and similar single-channel kinetics and conductance (∼60 pS) to the native receptors of lamina interneurons. In contrast, HisCl1 homomers, as well as heteromers, appear to be more sensitive to histamine (EC50 = 14 μm and 1.2 μm, respectively), with much lower single-channel conductance (∼4 pS) [19]. Despite the ionotropic histamine receptors in fruit flies being very different in terms of molecular structure and signal transduction mechanisms from the metabotropic receptors present in mammals, they share responsiveness to histamine and comparable pharmacology. Results obtained for *Drosophila* might thus provide strong implications for fundamental and translational cross-species research.

## 3. Histamine Metabolism

In the vertebrate CNS, the histamine levels are regulated by age, sex, and health/disease condition. In humans, histamine deficiency is traditionally associated with narcolepsy, sleep disorders, food intake, and, more recently, schizophrenia and pathological conditions, among which ischemia, traumatic brain/spinal cord injury, Alzheimer’s, Huntington’s, Parkinson’s diseases, amyotrophic lateral sclerosis, and multiple sclerosis [21,22,23,24]. Although some of these conditions do not find exact counterparts in *Drosophila*, others, such as photoreception, the wake–sleep cycle, food intake habits, or complex behaviors, including thermo/chemosensory reactions and courtship, are under histamine-regulatory mechanisms. Additionally, several multivariate pathological conditions can be reproduced in transgenic flies by expressing disease genes that are able to reliably reproduce human symptoms. For this reason, information acquired for *Drosophila* could likely be translated to higher organisms and provide potential therapeutic applications, even in humans. This might also be achieved by replacing mutations in genes related to histamine metabolism and signaling by gene therapy, with the potential to prevent, treat, and even cure several and severe pathological conditions.

**Figure 2:**
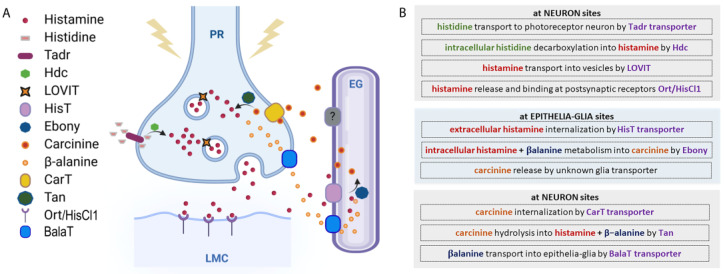
Histamine metabolic pathway. (**A**) Histamine metabolism. At the presynaptic terminal of the photoreceptor neuron (PR), histamine is synthesized by the Hdc enzyme from L-histidine, which is transported by Tadr. Histamine is transported into vesicles by LOVIT. After a stimulus, vesicles release histamine that binds Ort/HisCl1 receptors exposed by the large monopolar postsynaptic cells (LMCs). Histamine reuptake is mediated by a HisT transporter placed on epithelial glia (EG), where histamine is coupled with β-alanine and transformed into carcinine by Ebony. Carcinine exits the EG and returns to PR through the CarT transporter and is then hydrolyzed into histamine and β-alanine by Tan hydrolase. β-alanine is released by neurons and internalized by glia through the BalaT transporters. (**B**) Schematic view of histamine metabolism in *Drosophila*.

In order to preserve homeostasis and regulate the fine-tuning of specific normal and pathological pathways and functions, the extracellular/intracellular concentrations of histamine have to be strictly regulated. This occurs by modulating its synthesis, storage, release, and/or degradation mechanisms (Figure 2).

### 3.1. Synthesis

Histamine is synthetized intracellularly after the decarboxylation of the amino acid histidine by L-histidine decarboxylase (HDC), an enzyme whose expression is particularly enriched in mammal’s mast cells, basophils, enterochromaffin-like cells, and histaminergic neurons in the posterior hypothalamic area [25,26]. In *Drosophila*, ready to be used as a Hdc substrate, a huge amount of L-histidine is always transported and accumulated at photoreceptor terminals by Tadr (torn and diminished rhabdomeres), a plasma membrane transporter belonging to the cationic amino acid transporters superfamily [27]. Mutations in the *tadr* gene alter the synaptic transmission of photoreceptors and diminish the histamine axonal levels [28]. Histamine is next synthesized at presynaptic release terminals [29] via a single decarboxylation step from the amino acid L-histidine by the intracellular enzyme Hdc encoded by the *hdc* gene [30]. The Hdc enzyme is indeed present at very high concentrations in the presynaptic terminal (photoreceptor) in order to grant rapid and high-frequency visual signaling through robust de novo histamine synthesis. The null allele *hdc^JK910^* completely prevents histamine production and, when homozygously expressed, causes blindness in flies, further reinforcing the role of histamine in visual perception [31,32,33]. Interestingly, homozygous mutant *Drosophila* larvae are capable of reacting to light stimuli, demonstrating that larvae photoreceptors can use additional neurotransmitters [31], whereas adults use only histamine. Furthermore, the hypomorphic mutants *hdc^P211^*, *hdc^P217^*, and *hdc^P218^* show different levels of histamine and correspondingly different degrees of visual (and mechanosensory, see Section 4.5) impairments [31].

### 3.2. Storage, Release, Reuptake

After synthesis, histamine is stored and packed into intracellular granules/synaptic vesicles by LOVIT (loss of visual transmission), a vesicular histamine transporter [34,35]. In photoreceptors, the knockdown of LOVIT prevents visual synaptic transmission [34]. LOVIT is similar in mammals to the vesicular monoamine transporter-2 responsible for loading monoamines (not only histamine) into secretory granules [36]. Active extracellular release of histamine from the granules/vesicles then occurs by cell-specific mechanisms that, in mammals, include neurotransmission and/or wide diffusion through a concentration gradient. In fruit flies, histamine is released into the synaptic cleft by the photoreceptor or mechanosensory neurons that are depolarized, for instance, upon light, temperature, or mechanosensory stimuli. Histamine re-uptake from the synaptic cleft is then mediated by a recently characterized Na^+^-dependent transporter expressed by neighboring epithelial glia cells, HisT (histamine transporter) [37], while in mammals, it is mediated by the monoamine serotonin, dopamine, and norepinephrine transporters (Na^+^- and Cl^−^-dependent high-affinity uptake-system-1, or Na^+^- and Cl^−^-independent low-affinity uptake-system-2) [38].

### 3.3. Degradation

In vertebrates, free extracellular histamine can undergo oxidative deamination by diamine oxidase (an extracellular enzyme whose shortage in the human body causes allergy or histamine intolerance) [39], while intracellular histamine is metabolized by the cytosolic enzyme histamine-N-methyltransferase, which is not found in invertebrates and plants, and catalyzing the methylation of histamine in the presence of the S-adenosylmethionine. This is very different from what occurs in *Drosophila*, where histamine, after being removed from the synaptic cleft by the HisT transporter, is intracellularly inactivated in epithelial glia by conversion into carcinine after the binding with β-alanine mediated by the N-β-alanyl-dopamine synthase Ebony [40,41,42]. Carcinine then exits the epithelial glia cells through a still-unknown transporter and is in turn internalized into the photoreceptor synaptic terminal by CarT (carcinine transporter), an organic cationic shuttle. *Cart* gene deletion leads to blindness and strongly reduces the histamine amount, thus confirming the pivotal role of this transporter in the regulation of visual synaptic transmission [43,44,45]. Inside the photoreceptor neuron, carcinine is hydrolyzed back into histamine and β-alanine by the N-β-alanyl-dopamine hydrolase Tan [40]. Histamine is next stored in LOVIT-expressing vesicles (ready to restart the entire cycle and to be released again from the vesicles into the synaptic cleft after a stimulus), whereas β-alanine is released by neurons and internalized by glial cells due to the BalaT transporters [46] (Figure 2).

## 4. Histamine Functions

In mammals, histamine finds applications in multiple physiological functions, including circadian rhythms, eating behavior, mood, and learning, as well as in pathological conditions, such as allergy, obesity, gastrointestinal or neurological disorders, amnesia, and autoimmune diseases [21,22,47,48,49]. In *Drosophila*, histamine is similarly involved in various functions, in particular visual transmission [50,51,52], the sleep–wake cycle [53], temperature sensing [54], courtship behavior [55], and mechanosensory transmission [4,56].

### 4.1. Visual Transmission

**Figure 3:**
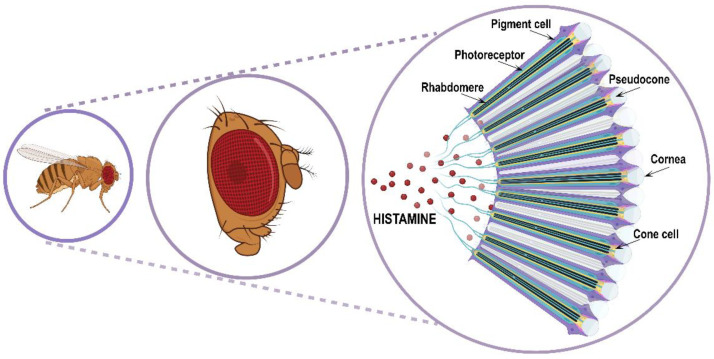
Schematic representation of *Drosophila* compound eye and ommatidia disposition.

Visual and spatial information processing allows animals to perceive the environment and consequently adapt their behavior according to circumstances such as escaping from predators or catching prey, finding food or mates, and sleeping or staying awake. For performing specialized tasks in visual and/or circadian behavior, *Drosophila* possesses three different types of eyes: two compound eyes placed on either side of the head capsule (Figure 3); three ocelli arranged in a triangle on the vertex of the adult head (dorsal head capsule) and between the compound eyes; and finally, two extraretinal Hofbauer–Buchner eyelets positioned between the compound eye and its associated optic ganglion.

The adult *Drosophila* compound eye originates during the larval and pupal stages from the neuroepithelial eye’s imaginal disc. The eyes of the larval stage are simpler and composed of only 12 photoreceptor neurons projecting to the larval optic neuropil compartment, where second-order neurons receive visual inputs [57]. Only the compound eye produces image-forming vision, as well as circadian photosensitivity. Image-forming vision in *Drosophila* initiates in the external visual organ, that is, the compound eye with the retina. Information about the color, shape, contrast, orientation, and motion of the surrounding objects is captured, fixed, and delivered from the retina to the optic lobe responsible for neuronal computation and processing, and which contains more than 60% of the total neurons of the brain [58]. The signals computed in these early visual areas are then conveyed by visual projection neurons to higher-order brain regions in the deepest part of the visual system, which are the optic glomeruli also called ventrolateral neuropil, where local interneurons and projecting neurons reliably decode visual informative subsets and convey task-performing information to the rest of the body [59,60,61]. The adult compound eye is composed of the retina and optic lobe.

The retina is an assembly of about 700/750 (respectively in male/female) repeating hexagonal units called ommatidia. Each ommatidium is a unit of 19 cells consisting of 8 rhodopsins expressing R1–R8 photoreceptor histaminergic neurons (turquoise structure in Figure 3) converting light into electric signals and 11 accessory pigment cells (purple structure in Figure 3) optically insulating each ommatidium from its adjacent neighbors [12,62,63]. Overlying the eight photoreceptor neurons, there is a quartet of non-neuronal cells (Semper cells or cone cells, beige structure in Figure 3) sharing features with vertebrate retinal Muller cells and forming the base of a cavity (pseudocone, grey structure in Figure 3), where they secrete the extracellular fluid that constitutes the corneal lens [64]. The pseudocone is capped by a dioptric chitinous cuticle, the cornea (gray structure in Figure 3). R1–R6 photoreceptor neurons lie in a hexagonal ring and project to the lamina in the optic lobe, and are responsible for achromatic light-sensing image formation and motion detection, while R7 and R8 are central within the R1–R6 ring, project to the medulla in the optic lobe, and are responsible for color-sensing and light polarization functions. Photoreceptors are highly polarized neuroepithelial cells possessing two types of specialized membrane compartments. The apical light-sensor compartment, the rhabdomere (black structure in Figure 3), consists of a tightly dense stack of 30–50 thousand microvillar structures with visual pigments, while the basolateral membrane extends into an axon. The R1–R6 photoreceptor cells express the major rhodopsin Rh1, the R7 photoreceptors express either Rh3 or Rh4, and R8 cells express either Rh3, Rh5, or Rh6 [65]. Importantly, rhodopsin mutations and trafficking defects are recurrent causes of retinal degeneration in flies, as well as in mammals.

The optic lobe is formed by four ganglia/neuropils, named the lamina, medulla, lobula, and lobula plate, arranged as repetitive structures with several hundred retinotopical columns for the parallel processing of visual inputs from different space points. It contains both interneurons whose cell bodies and projections remain within the optic lobe, and projection neurons connecting the optic lobe to the central brain, particularly to the ventrolateral neuropil (optic glomeruli).

The lamina is the first optic ganglion in the optic lobe and is arranged in radial synaptic units (cartridges). It receives inputs directly from the retina and is devoted to light contrast enhancement. The lamina contains several different classes of neurons: distinct L1–L5 lamina monopolar cells that are output neurons sending axonal processes to the medulla and corresponding to the bipolar cells of the vertebrate visual system; C2- and C3-GABA-ergic feedback neurons that are presynaptic on several cell types in the lamina; Lawf1 and Lawf2 wide-field feedback neurons that transmit from the medulla back to the lamina; lamina-intrinsic amacrine neurons that are uniquely confined to the lamina; T1 basket cells that consist of feedback neurons with cell bodies in the medulla and axons in the lamina; and finally lamina tangential neurons that arborize in the ipsilateral central brain [66,67]. Both the lamina and medulla decode information from small areas of the visual field after facing an anteroposterior inversion between the lamina and medulla through the first optic chiasm.

The medulla represents the second ganglion in the optic lobe and is distinguished along the horizontal axis by 10 different M1–M10 compartments, or layers, connected to photoreceptor and lamina neurons by stereotypic patterns. Similar to the lamina, the medulla is organized in columns, each containing a fixed number of columnar neurons (17 medulla cells and 10 terminal inputs from the lamina projecting to the medulla) [68].

The lobula complex contains the third and fourth ganglia and represents the most sophisticated visual neuropil in the optic lobe. It is composed of the lobula plate, a tectum-like structure, and the lobula, a cortex-like structure containing columnar neurons resembling mammalian pyramidal neurons in the striate cortex (the primary sensory cortical area for vision in mammals). The lobula complex can precisely identify object shapes by integrating visual information not only from the whole retinal input, but also from the entire visual field comprising that originating from the ocelli. The supraesophageal (i.e., the brain) and subesophageal (roughly resembling the spinal cord of vertebrates) ganglia are the principal output regions of the optic lobe.

The optic glomeruli (also named ventrolateral neuropil) in the adult brain of *Drosophila* are prominent neuropil structures enriched for synaptic sites and intense neurite interconnectivity, where local interneurons and visually projecting neurons from the optic lobe (particularly from the lobula complex columnar neurons) decode visual primitives. They are positioned in the ventrolateral central brain, in regions called the posterior ventrolateral protocerebrum and posterior lateral protocerebrum. Also, the dorsal brain region named the anterior optic tubercle is considered an optic glomerulus [69,70].

The adult ocelli are characterized by cone cells, pigment cells, and about 80 extraretinal photoreceptor neurons expressing the Rh2 violet-sensitive rhodopsin and directly innervating the lobula complex in the optic lobe. The ocelli are characterized by single optical systems, different from the compound eye that consists of multiple ommatidial optical systems, as described above. Given that the lobula and lobula plate in the optic lobe receive visual information originating from the ocelli, other than from the retina of the compound eye through the lamina and medulla, the global visual information transmitted to the deepest structures of the central brain is an integration of information from both the ocelli and compound eyes. Overall, the ocelli augment those visual impulses sustaining non-image-forming behaviors, such as the phototaxis, color perception, flight stabilization, and circadian habits that are normally governed by the compound eye.

The Hofbauer–Buchner eyelets containing pigment granules and numerous microvilli arranged in rhabdomeres positive for Rh6 green-sensitive rhodopsin, participate in the determination of the molecular clock and circadian photoreception through the compound eyes. As a matter of fact, extraocular photoreceptors do not perform complex image analysis; rather, they are involved in circadian rhythms and photoperiodic responses.

Anatomically speaking, light signals in flies may be processed by visual and non-visual structures: compound eyes with their ommatidia and the extraretinal Hofbauer–Buchner eyelets photoreceptors are immunoreactive to histamine and are included in the first category, whereas the second group concerns the ocelli and the deep brain circadian photoreceptors that are not visual structures [71]. By combining genetics with immunohistochemistry and electrophysiology approaches, in the past two decades, it has been possible to outline almost all the players involved in the visual neurotransmission pathway of adult *Drosophila* [72]. As mentioned above, upon light stimulation of photoreceptors, the canonical inhibitory fast neurotransmitter histamine is released to the postsynaptic neurons. This causes the hyperpolarization of the postsynaptic membrane and thereby a decrease in neuronal activity with an increase in the action potential threshold. While the larval photoreceptors employ a transmitter(s) totally different from histamine, it is notable that, in the adult compound eye, in addition to histamine, acetylcholine (ACh) has recently been shown to transmit to a specific subclass of photoreceptors [73,74]. Indeed, it has been demonstrated that the postsynaptic partners of the inner R8 photoreceptors that terminate in the medulla respond to ACh other than histamine by possessing a dual transmitter phenotype and a differential expression of both transmitter receptors [73]. Moreover, R8 photoreceptors possess the ACh-synthesizing enzyme choline acetyltransferase and the vesicular ACh transporter. Conversely, the choline acetyltransferase was not found in R1–R7 photoreceptors, and neither were neurotransmitters such as GABA, glutamate, serotonin, and dopamine, thus confirming the exclusive histaminergic nature of R1–R7 photoreceptors [74]. Finally, it was recently demonstrated that synaptic ACh signaling from lamina-intrinsic amacrine neurons, as well as lamina L4 interneurons, facilitated the rapid repolarization of L1 and L2 interneurons that are necessary for the termination of the photoresponse in photoreceptors and downstream laminar interneurons. This also indicates that, in addition to histamine, parallel ACh neurotransmission is necessary to maintain the response of *Drosophila* to high-frequency light stimulation [75].

### 4.2. Wake–Sleep Cycle

Light contrast-based mechanisms drive image-forming perception, while light irradiance-based mechanisms drive circadian photoentrainment. Two distinct photoreceptor populations are devoted to these tasks, but it is still unknown how photoreceptors can segregate irradiance–circadian photoentrainment from contrast-image signals. The regulation of the wake–sleep cycle is mediated by light irradiance inputs. Several sets of circadian clock neurons (an overall number of about 150 neurons in the CNS, classified by their size and location in four lateral and three dorsal groups) cooperate to orchestrate the activity rhythms of flies. They are distributed within numerous tissues, thus suggesting that the visual system primarily participates in circadian photoreception, but also that other elements are likely to be involved. While the compound eye is devoted to both image perception and circadian photoreception, ocelli and Hofbauer–Buchner eyelets make only a limited contribution to these functions. The circadian system uses multiple photoreceptors to connect light/dark perception to photoperiodic activity in fruit flies, but how the photoreceptors’ information is finely integrated into the clock neuron network is not yet clear. If visual perception in *Drosophila* finds histamine as almost the only player, the wake–sleep cycle is conversely regulated by the interaction of multiple neurotransmitters. In particular, there are sleep-promoting neurotransmitters (serotonin and GABA), wake-promoting ones (histamine, dopamine, and octopamine), and those that may serve both functions (glutamate and ACh). For a more comprehensive review, please refer to [74,76]. Concerning the neurotransmitters associated with wakefulness, while dopamine and octopamine inhibit the activity of the sleep centers [77], histamine is mostly involved in the connection between photoreceptors (retinal and extra-retinal) and clock neurons [53]. In particular, the *Drosophila* R8 photoreceptor’s transmission segregates image perception from irradiance photoentrainment signals at the first-order synapses of the visual system by co-releasing two neurotransmitters, histamine and ACh. This segregation also occurs at the postsynaptic level in the medulla through histaminergic input from a single R8 photoreceptor to each unicolumnar neuron, and through the integration of cholinergic inputs from up to 100 R8 photoreceptors to each accessory multicolumnar arcuate integration neuron, which, in turn, directly excites downstream clock neurons.

During the light phase of the light–dark cycle, the histamine HisCl1 receptor mediates an autocrine negative feedback of histamine release in R8 photoreceptors that, in turn, increases ACh release and maintains continuous irradiance signals to clock neurons that control the rest–activity rhythms [73,74]. However, R1–R6 photoreceptors can also use histamine to excite circadian clock neurons, although it remains to be explained how R1–R6 photoreceptor neurons might be connected to R8 cells. In *Drosophila*, the clock neurons synchronize with the circadian cycle, either through cryptochromes (a class of blue-light photosensory flavoproteins regulating light entrainment) or rhodopsin–histaminergic photoreceptors. A recent study has established that both Ort and HisCl1 histamine receptors mediate the cryptochrome-independent pathway of the rest–activity cycle and contribute to its synchronization, but act on two different neuronal populations, the optic lobe interneurons that are involved in visual functions and the Rh6-expressing R6 photoreceptors. Interestingly, Ort functions cannot be rescued by *HisCl1* expression in *ort*-expressing cells, whereas Ort activity can rescue HisCl1 function in R6 photoreceptors [12]. 

The Hofbauer–Buchner eyelets play a precise role in the circadian clock neuron network by synergizing cryptochrome-mediated photoreception in the small ventral lateral neurons and antagonizing cryptochrome- and compound-eye-based photoreception in the HisCl1-positive and histamine-receptive large ventral lateral neurons. In addition, histamine stops spontaneous firing on these last neurons [78]. Finally, the release of histamine promoted by a light stimulus sustains two different pathways related to the sleep–wake cycle: the photic pathway and that associated with motion detection, both involving the activation of lamina interneurons [79,80]. Interestingly, both in flies and mammals, the administration of histamine promotes wakefulness and sleep reduction [53,81]. Mutations on the *hdc* gene determine sleep disturbances and, more precisely, an increase in sleep duration, whereas HisCl1, but not Ort mutants, causes long-sleep phenotypes [53]. Consistently, *HisCl1* mutants with an increased sleep duration can be rescued by the overexpression of the receptor in circadian clock neurons expressing the neuropeptide pigment dispersive factor, thus demonstrating that the histamine–HisCl1 axis regulates the wake/sleep cycle and that wake signals travel through neuropeptide pigment dispersive factor-sensitive neurons [53].

Interestingly, histamine also modulates the wake–sleep cycle in higher organisms. For instance, pitolisant (H3R receptor antagonist/inverse agonist) is a narcolepsy treatment option [82,83] and modafinil (a drug increasing histamine release in the neocortex and hypothalamus [84,85]) is a wake-promoting agent to treat hypersomnolence [86,87]. These results certainly encourage further research on the role of histamine in the sleep–wake cycle and circadian rhythm regulation. We are confident that further exploitation of circadian rhythms modulated by histamine in *Drosophila* might uncover novel pathways and consequently identify additional drug treatments for narcolepsy and sleep disturbance.

### 4.3. Temperature Preference

Temperature is perceived on the body’s surface in *Drosophila* by both primary thermosensory neurons in the PNS (located at the base of the arista, i.e., the terminal segment of the antenna). These neurons project to a central region defined as the proximal antennal protocerebrum, where secondary thermosensory neurons (named thermal projection neurons) in turn project to the brain, and particularly to the lateral protocerebrum, the mushroom bodies, and the lateral horn, where thermal information processing is achieved. At the molecular level, cold- or heat-sensitive TRP (transient receptor potential) channel families, gustatory receptor GR28B(D), a heat-sensitive protein, and additional ionotropic receptors have been identified as thermal sensors in both larval and adult *Drosophila* [88]. Histamine is known to regulate temperature preference by signaling through its Ort and HisCl1 receptors. However, the fruit fly genome sequencing project (https://www.genome.gov/11008080/fruitfly-genome-sequencing, accessed on 15 April 2024) anticipates the presence of a novel ligand-gated chloride channel sensitive to pH as well as to histamine, named pHCl, with almost identical similarity to histamine-, glutamate-, and glycine-gated ion channels, but without strictly belonging to any of these ion channels [89]. Remarkably, currents through this pHCl channel are found to be induced by elevated temperatures, thus providing the first indirect evidence for histamine-dependent temperature sensing in *Drosophila*. This novel pHCl channel is present in embryos, larvae, pupae, and adults. In situ hybridization has, moreover, demonstrated the expression of pHCl in the embryo neural cord and hindgut. The pHCl receptor channel is inhibited by extracellular protons and activated by avermectins (blocking electrical transmission in invertebrate nerves and muscle by enhancing the effects at the glutamate-gated chloride channel) in a pH-dependent manner, demonstrating the unique pharmacological and biophysical features of this channel [89].

A more direct role for histamine and its ionotropic receptors in thermoregulation was next demonstrated by Hong et al. [54]. In order to identify genes involved in temperature sensing, the authors screened a huge number of independent P-element insertion mutants of *Drosophila* and found that defects in the genes coding for histidine decarboxylase and histamine receptors HisCl1 and Ort caused abnormal temperature preferences. The histaminergic mutants possessed a reduced tolerance for high temperatures and enhanced tolerance for cold. Remarkably, these abnormal temperature preferences could be restored by genetic rescue, but were also reproduced in wild-type flies by the administration of the antagonists for histamine receptor cimetidine (H2R) and hydroxyzine (H1R). These data also demonstrate a clear role for histamine signaling in the modulation of temperature tolerance and preference.

Two *Drosophila* mutants of the histamine-gated chloride channel hisCl1, named hclB(T1) (P293S) and hclB(T2) (W111*, a null mutation), were finally studied for temperature sensing, in particular for their lapse into, and recovery from, paralysis induced by a high temperature of 41 °C. In particular, the hclB(T2) flies went into a coma faster than the hclB(T1) or wild-type flies, and both mutants recovered more slowly, therefore corroborating the histamine-dependent temperature preference in fruit flies [90].

### 4.4. Courtship Behavior

Courtship and mating are complex and innate behaviors under the control of apparently a single gene, named *fruitless*, which is necessary and sufficient for the distinct phases of the entire ritual in *Drosophila* that is under the control of histamine, among other agents. It was recently reported that the gene *Trapped in endoderm 1* (*Tre1*), an orphan receptor signaling via multiple heterotrimeric G-proteins, was a new gene expressed with sexually dimorphic patterns in CNS and PNS neurons, and was involved in normal courtship and mating behavior in *Drosophila* [55]. Of note, Tre1 is required for cell polarity and migration. Tre1 responds to histamine as a natural agonist, and this is quite surprising, representing the very first evidence for active metabotropic histamine receptors in *Drosophila*, in addition to the long-established histamine-activated chloride channels Ort and HisCl1. This would reinforce a clear role for histamine in mate identification and courtship [55].

### 4.5. Mechanosensory Transmission

By interfering with functions such as crawling, walking, jumping, flying, hearing, and mating, mechanosensory transmission is a central process in *Drosophila*’s daily life. Indeed, to properly cope with the environment and trigger the correct behavior, fruit flies need to not only integrate sensory and motor stimuli, but also to distinguish between inside and outside inducements. To this end, the CNS exploits the so-called corollary discharge circuits, which deliver predictive motor information to correctly trigger sensory and motor systems. Interestingly, a recent study provided the anatomical and physiological characterization of two pairs of ascending neurons belonging to corollary discharge circuits and using histamine as a neurotransmitter. These ascending histaminergic neurons originate in the ventral nerve cord and project to the brain, properly conveying the external sensorimotor stimuli in order to finely tune the appropriate motor response [56].

The perception of mechanical stimuli and tactile reflexes starts when specialized mechanosensory neurons convert mechanical forces into electrical signals that are translated into activity patterns within the primary and secondary mechanosensory regions of the brain. The Johnston’s organ, located inside the antenna, is the largest mechanosensory organ in fruit flies and contributes to touch, proprioception, hearing, and wind sensing. It contains mechanosensory neurons that are located in the second segment of the antenna and that are sensitive to the rotations of the distal segment of the antenna [91]. Anatomically and physiologically distinct mechanosensory neuron subpopulations in the Johnston’s organ perceive diverse mechanical forces and influence different behaviors by projecting to distinct and discrete regions of the brain. Each mechanosensory subpopulation elicits antennal grooming, but distinct subpopulations influence, for instance, the additional task of wing flapping or backward locomotion. Although mechanosensory neurons are heterogeneous to allow specific sensory functions, they also share common structural features and transduction molecules [92]. In fruit flies, histamine is involved in mechanosensory transmission and, consequently, histamine is detected in specific mechanosensory neurons of cuticular hair sensilla, and in a few neurons in the head and body ganglia [4]. In particular, all mechanosensory neurons of hair sensilla are positive for histamine staining in their cell bodies, axons and fiber bundles in peripheral nerves, and terminal projections in the central neuropil of the head and thoracic ganglia. Histamine staining is absent from other mechanosensory organs, such as campaniform sensilla (distributed in the cuticula across the legs, antennae, wings, and halters, and innervated by the dendrites of a single bipolar sensory neuron), and scolopidial organs (a segmental stretch proprioceptor in the PNS, formed by three glial cells and one bipolar neuron) [4].

Mutant flies deprived of the *hdc* gene (see Section 3.1) or producing decreased amounts of histamine (alleles *hdc^P211^*, *hdc^P217^*, and *hdc^P218^*) exhibit various degrees of mechanosensory impairment (other than visual) and inefficient grooming of the body surface, as evaluated by quantitative behavioral assays [31]. When these mutant flies are fed with aqueous histamine or are genetically rescued by a transgene ubiquitously expressing *hdc*, sufficient amounts of histamine are accumulated in mechanoreceptor neurons (as in photoreceptor neurons) to generate near-normal electrical responses able to restore mechanosensory behavior, such as grooming or bristle-scratch reflexes [32].

## 5. Histamine Sensing

All animals depend on taste sensors to evaluate food sources. Taste perception is fundamental for avoiding toxic and lethal food, thus preserving animal wellbeing. In *Drosophila*, gustatory sensilla precisely devoted to taste perception are abundant on the proboscis (labellum and esophagus), segments of the legs, ovipositor, and margin of the wings.

There are three types of taste sensilla in the different taste locations: taste bristles, taste pegs, and pharyngeal hairless sensilla. The labellum is the main taste organ on the fly’s head containing 31 taste sensilla, each with a pore at the tip. Three types of sensilla exist: large—L, intermediate—I, and small—S. Sensilla are innervated by one mechanosensory neuron, in addition to 1–4 gustatory neurons (namely for sweet, bitter, salt, and water) carrying gustatory receptors belonging to a gene family with 60 members, encoding 68 different receptor proteins. The activation of these receptors delivers environmental information to the brain that accordingly prompts taste acceptance or avoidance behaviors [93,94,95]. *Drosophila* can discriminate histidine from histamine using S-type sensilla carrying bitter-sensing gustatory neurons in the labellum. Through electrophysiological studies, it was shown that histamine activates GR22e and IR76b gustatory receptors that are necessary, but not sufficient, for histamine sensing. The ectopic expression of GR22e induces a response in I-type sensilla that are not usually histamine-sensitive [96]. In a further study, some previously uncharacterized gustatory receptor genes were screened by RNA interference and examined by electrophysiological and behavioral tests, such as binary food choice and proboscis extension response. Newly generated null mutants and recovery experiments after wild-type cDNA expression in bitter-sensing gustatory neurons demonstrated that GR22e, GR9a, and GR98a are specific histamine-responding receptors. Moreover, it was established that histamine sensing was apparently mediated by the labellum, but not by sensilla residing in the legs, as demonstrated by the proboscis extension response assay [97]. By investigating histidine and histamine taste perception, it was further shown that high levels of histamine could become toxic to flies by inducing strong avoidance responses. Given the fly’s gustatory aversion to histamine, for pharmacological purposes, it might be advisable to administer histidine to fruit flies instead of histamine in order to prevent bitter sensing and consequent pharmacological avoidance [44].

## 6. What Next

*Drosophila* is a very simple, but versatile, model organism copiously assisting researchers in untangling normal functions and predicting pathological derangements occurring in higher organisms, including humans. Histamine has been one of the most exploited molecules in biomedical research for several years, with actions ranging from sleep, arousal, and circadian rhythmicity to a central role in the pathogenesis of several allergic and neurological conditions. We have centered our review on the knowledge that the use of *Drosophila* has added to the already vast histaminergic field. We have described histamine’s actions on photoreceptors sustaining the visual system and synchronizing circadian rhythms, on temperature preference, courtship behavior, taste perception, and mechanosensory transmission. Moreover, we have highlighted the functional consequences of mutating genes involved in histamine metabolism and signaling. Based on the rapid development of increasingly sophisticated investigative technologies and the easy access to artificial intelligence/computation analysis, we expect that the field might now proceed even further, for instance with: (i) a re-evaluation of histamine receptor–ligand cross-talk using more appropriate state-of-the-art methodologies; (ii) an optimization and standardization of new parameters and criteria for novel histaminergic ligand development and experimental model selection; (iii) a clearer consideration of species/strain discrepancies, with the aim of translating histamine preclinical data into clinical settings; and (iv) a broader disclosure of histamine-related clinical trials results. In this way, we expect to: (i) promote a complementary, cross-methodological, and multidisciplinary approach to further dissect histaminergic signaling and functions; (ii) deliver beneficial histaminergic endpoints through basic and translational research; and (iii) define the precise stage–gate process for the development of active and safer histaminergic therapeutic options against widespread and debilitating diseases. Indeed, given the high impact of histaminergic physiology in *Drosophila* and the partial similarity with mammals, fruit flies are prone to be considered a very strong system for pursuing at least part of his goal and analyzing the potential effect of histaminergic compounds, particularly in pathological backgrounds. This will sustain a constant effort in translating preclinical results into clinical options against the most widespread pathological conditions where histamine elicits major effects [98]. Indeed, in addition to playing a central role in the pathogenesis of several allergic diseases and inflammation, histaminergic signaling now exhibits a clear involvement in the nervous system, with interesting roles demonstrated in clinical conditions such as Parkinson’s and Alzheimer’s disease, schizophrenia, dementia, depression, cerebral ischemia, multiple sclerosis [21,23], and amyotrophic lateral sclerosis [22,99,100,101]. In particular, the histaminergic compound pitolisant (Wakix^®^, H3R inverse agonist) is being tested in Phase-III clinical trials for drug abuse and sleep disorders and has been approved for narcolepsy by the European Medicines Agency. Moreover, hydroxyzine (H1R antagonist), the compound AVN-101 (H1R ligand), GSK247246, and GSK239512 (H3R inverse agonists) are under clinical testing in patients with relapsing–remitting or progressive forms of multiple sclerosis [23]. Finally, recent studies have identified new histamine–ALS and –MS drug associations, also predicting off-label novel uses of histaminergic drugs for ALS [99] and MS [102] diseases.

In conclusion, by hopefully promoting critical discussion and further research, with our work, we emphasize and renew the importance of histaminergic research in biomedicine through the exploitation of *Drosophila*, and likely extend the scientific debate to the academic, industry, and general public audiences. Despite its wide use in the scientific community, research focused on *Drosophila* is still quite unfamiliar to or underestimated by the general public. By reporting on basic and translational histamine research performed based on *Drosophila* genetics, metabolism, physiology, and behavior, we expect to increase the awareness and appreciation of this invertebrate organism and convey its potentialities also to non-specialists.
